# Histopathological, Immunohistochemical and Biochemical Studies of Murine Hepatosplenic Tissues Affected by Chronic Toxoplasmosis

**DOI:** 10.1155/2022/2165205

**Published:** 2022-06-16

**Authors:** Samah Hassan Yahia, Samia Elsayed Etewa, Nesreen Saeed Saleh, Samira Metwally Mohammad, Nora Ibrahim Aboulfotouh, Ahmad Mansour Kandil, Mohamed Hassan Sarhan

**Affiliations:** ^1^Medical Parasitology Department, Faculty of Medicine, Zagazig University, Egypt; ^2^Zoology Department, Faculty of Arts and Sciences Al-Wahat, Benghazi University, Libya; ^3^Medical Parasitology Department, Faculty of Medicine, Mansoura University, Egypt; ^4^Pathology Department, Faculty of Medicine, Al-Azhar University-Cairo, Egypt

## Abstract

Toxoplasmosis is a serious health problem in humans and animals resulting from obligatory intracellular invasion of reticuloendothelial tissue by *Toxoplasma gondii*. The profound pathologic effect of toxoplasmosis is confined to nervous tissue, but many other organs, including the liver and spleen, are insulted. Many molecules like caspase-3, CD3, and CD138 are implicated in the tissue immune response in a trial to alleviate hazardous toxoplasmosis impact. This study aimed to investigate the effect of chronic toxoplasmosis on the liver and spleen tissues of mice using biochemical and histopathological techniques and to detect the activity and level of expression of caspase-3, CD3, and CD138 in these tissues using immunohistochemical labeling. Compared with normal control, altered normal histological features accompanied by inflammatory reaction were recorded in hepatosplenic reticuloendothelial tissues in chronically infected mice. The biochemical profile of the liver has been changed in the form of increased liver enzymes, and oxidative stress has been evidenced by elevated nitric oxide (NO) concentration in liver homogenate. The levels of caspase3, CD3, and CD138 were markedly expressed in the liver and spleen of infected mice. Our findings revealed the persistent effect of latent toxoplasmosis on the host's histological architecture, metabolic, and immunological profile, creating a continued challenging host-parasite relationship.

## 1. Introduction

Toxoplasmosis is a serious health problem in humans and animals due to obligatory intracellular invasion of reticuloendothelial tissues by a protozoan parasite called *Toxoplasma gondii* [1, 2]. Primary infection with toxoplasmosis occurs due to contamination of food or drink by tissue cyst, pseudocyst, or cat oocyst [3, 4]. Secondary infections are less commonly noticed in congenital toxoplasmosis or transplantation of organs due to exacerbation of chronic latent disease [5].

The pathological effect of toxoplasmosis is not confined only to nervous tissue and can affect many other organs, including the liver and spleen of the host [6]. Although the parasite spreads early to the liver in the early course of acute infection, no significant clinical alterations or laboratory abnormalities can be detected [7]. However, in some cases, many pathological modifications in the liver can occur according to strain virulence, in the form of hepatomegaly, hepatitis with altered liver function tests [8–10], cholestatic jaundice [11], and cirrhosis [12]. In liver transplant recipients, toxoplasmosis can cause liver dysfunction [13] that may progress to acute liver failure and transplant rejection [14].

During *Toxoplasma* infection, many molecules are secreted to maintain a continuous interaction condition between the parasite and the immune system in an ongoing effort to elude the host's defensive response [15]. TNF-*α*, interleukin-6 (IL-6), and IL-12 as innate immune molecules activate natural killer cells (NK cells) and both CD4+ T and cytotoxic CD8+ T cells subsets. This activation ends up with the production of a considerable amount of IFN-*γ*, which is essential in the host defense response against *T. gondii* parasite [16]. Many other molecules like caspase-3, CD3, and CD138 could be implicated in the immune response against toxoplasmosis.

Caspase-3 is a cysteine-aspartic acid protease protein belonging to a caspases family that harbors both a cysteine and histidine residue on their active site [17]. The primary function of caspase-3 is well documented in programmed cell death (apoptosis) through activation of both intrinsic (mitochondrial) and extrinsic (death ligand) pathways [18, 19]. This activation ensures a controlled cell demise with minimal proteolytic effects on the surrounding tissues [20]. However, this role in cell death can be inhibited by some infections like toxoplasmosis, giving it a chance for long-lived infection in host tissue [21]. Caspases' critical involvement in cell death and disease has prompted research into exploiting caspases as a therapeutic target against various infections.

The cluster of differentiation 3 (CD3) is a unique T cell surface protein complex formed of four polypeptide chains called epsilon (*ε*), gamma (*γ*), delta (*δ*), and zeta (*ζ*). These chains pose negatively charged aspartate residues associated with the positively charged T-cell receptor (TCR) forming the TCR complex [22]. This complex is involved in intracellular signaling and the expression of TCR surface molecules [23]. CD3 is highly expressed on the surface of mature and all stages of T-cells, and nearly no other cells. This specificity makes this protein a helpful metric for determining the efficiency of an immune reaction in response to any stimuli, as well as a powerful T-cell (anti-CD3 antibodies) marker for identifying normal and dysfunctional T cells in tissues using immunohistochemistry labeling [24, 25]. CD3 is essential for T cell activation, which is critical to combat any foreign pathogens, so any drug that targets this molecule (anti-CD3 antibodies) is considered an immunosuppressant agent [26].

CD138 (Syndecan 1) is a heparin sulfate proteoglycan protein and belongs to the four-member syndecan family. This protein binds the plasma cell membrane making it a good plasma cell marker in humans [27]. CD138 is highly expressed in fibroblasts and epithelial cells in an experimental animal model. Deficiency in this protein is linked to increased inflammation in various tissues, showing that this protein's primary function is anti-inflammatory [28–30].

Caspase-3 inactivation in host cells has been observed during toxoplasmosis [21]. This blockade prolongs the life of infected human neutrophils and prevents the activation of apoptotic caspases in infected cells, ensuring long-term infection in the host [31]. On the other hand, CD3 subsets have been activated, and their numbers increased in the peripheral blood and spleen of immunized and chronically infected mice [32]. In the sera of women infected with *T. gondii*, CD3 T cells were significantly upregulated compared to healthy controls, indicating that the lymphocytes are proliferating rapidly because of exposure to the *Toxoplasma* antigens [33]. The threshold of activation and differentiation of CD138 plasma cells is lowered during *Toxoplasma* infection [34].

The pathogenic effects of *T. gondii* extend to visceral organs, including the liver and spleen. The discovery of novel host liver molecules involved in the replication process of *T. gondii* opens the door to the development of new and more effective anti-*T. gondii* drugs. The purpose of this work was to investigate the activity and level of expression of CD3, CD138, and caspase-3 in murine hepatic and spleen tissues experimentally affected by chronic *T. gondii* infection.

## 2. Materials and Methods

This experimental case control study was carried out from October 2020 to March 2021 at the Parasitology Department, Faculty of Medicine, Zagazig University, Egypt.

### 2.1. Ethical Statement

Based on the international animal care rules, current mice investigation steps, including therapy and euthanasia, are approved by (IACUC) of Zagazig University. Mice were kept and raised in a pathogen-free animal facility at our department under convenient circumstances. All surgical procedures were conducted under isoflurane anesthesia, and every effort was made to ensure that the animals suffered as little as possible.

### 2.2. Parasite Strain

ME49 (cyst-forming type II) of avirulent chronic *Toxoplasma* strain was obtained from the Medical Parasitology Department, Faculty of Medicine, Alexandria University, Egypt.

### 2.3. Animal Infection

As blood tachyzoites and congenital diseases might occur during prolonged illness without any external source, all mice involved at the beginning of this study were serologically proved to be negative for toxoplasmosis [35, 36]. The infection was maintained by repeated inoculation in clean lab-bred Swiss albino mice. 20-25 gram weight and ten-week-old male mice were used to avoid problems associated with pregnancy and giving birth. Homogenized dissected brain tissues in 2 mL sterile phosphate-buffered saline (PBS) had been obtained from chronically infected euthanized mice. Cerebral tissue cysts were counted in two drops from the brain homogenate using a light microscope. The infective dose was adjusted to be 20 cysts/0.2 ml/mouse before being introduced by esophageal syringe to mice.

### 2.4. Experimental Design

All mice were divided into two experimental groups at random: group (A) (*n* = 20) mice were used as a healthy control group. In contrast, group (B) (*n* = 42) mice were infected orally with 20 tissue cysts of *T. gondii* avirulent strain (ME49) in 0.2 mL sterile PBS. Both animal groups were housed in plastic cages with white wood chips for bedding and free access to complete commercial meal combinations and tap water for drinking. They were also kept under-regulated lighting (12 h light/12 h dark cycle) and temperature (25 ± 2°*C*). Throughout the experiment, all mice were monitored twice daily to develop clinical manifestations and survival. Weight loss was also tracked as an indicator of morbidity. The efficacy of the experimental *Toxoplasma* infection in mice was validated four weeks post-infection (PI) by a complement fixation test to detect specific anti-*Toxoplasma*-IgM with the exclusion of animals with negative IgM levels. Nine weeks post-infection (wpi), the experiment was terminated as planned, and all mice were sacrificed to perform the intended assessment procedures. Isoflurane was used to euthanize all animals, including controls, followed by thoracotomy. After collecting blood from the chest cavity, all mice were killed by cervical dislocation. Immediately after collection, 1 ml blood was placed in a serum separator tube for biochemical examination. Each mouse's liver and spleen were promptly removed, chopped into tiny pieces, washed with saline solution (0.9% NaCl w/v), and preserved separately in 10% buffered formalin in 0.1 M phosphate buffer in preparation for histological examination and immunohistochemical studies (presence or absence of CD3, CD138, and caspase-3 immune reactive cells).

### 2.5. Biochemical Examinations

1 mL of blood was drawn through cardiac puncture and allowed to clot for at least 20 minutes before being centrifuged at 4 000 rpm for 5 minutes to extract the serum. The sera were kept at -20 °C. To examine the function of the livers of the control and infected groups, the activities of serum aspartate transaminase (AST), alanine aminotransferase (ALT), gamma-glutamyl transferase (GGT), thymidine phosphorylase (TP), and alpha-fetoprotein (AFP) were measured. All blood biochemical parameters were determined by automated photometric systems [37]. Nitric oxide (NO) levels in liver tissues have been evaluated colorimetrically as nitrite using the Griess reaction [38].

### 2.6. Histopathological Examination

Fresh liver and spleen tissue samples were submersed for one week in 10% neutral buffered formalin for fixation. After washing with tap water, successive alcohol dilutions (methyl, ethyl, and pure ethyl) were utilized for dehydration. Specimens were washed in xylene and stored in a hot air oven for 24 hours submersed in 56-degree paraffin. A microtome was used to make 5 *μ*m thick sections. The tissue micro-sections acquired were deparaffinized on glass slides and then stained with hematoxylin and eosin (H&E) dye [39]. An experienced pathologist blindly examined stained sections using a light microscope (Olympus, Japan) equipped with a high-resolution digital camera. Acute inflammation was characterized by neutrophil infiltration, whereas the presence of mononuclear cells defined chronic inflammation.

### 2.7. Immunohistochemical Staining (IHC)

A microtome was used to cut micro-sections of formalin-fixed paraffin-embedded (FFPE) livers and spleens. Three slices of each tissue were cut and mounted on glass slides. All immunohistochemistry tests were carried out in accordance with the protocol of a commercial streptavidin-biotin kit (Thermo Fisher Scientific Inc.) that employed diaminobenzidine (DAB) as a chromogen and Mayer's hematoxylin as a counterstain. Mouse tissues experimentally infected with the *T. gondii* ME49 strain served as a positive control for *T. gondii* antigens. Polyclonal rabbit anti-human CD3, monoclonal mouse anti-human CD138, and rabbit polyclonal antibody to caspase-3 (all diluted 1 : 100) were primary antibodies (DAKO, Glostrup, Denmark) to marker T-cells, B-cells, and normal tissue structure, respectively.

Immunostaining methods were carried out after clearing off paraffin by xylene and then rehydrating with decreasing percentages of ethanol in distilled water. After antigen retrieval, 3% peroxide hydrogen solution treatment blocked endogenous peroxidase activity in 10 ml citrate buffer (pH 6.0). Using 1% bovine serum albumin (BSA) in 0.1 M phosphate buffer saline (PBS) as blocking solution, the paraffin fixed sections were blocked and then stained with a mouse polyclonal anti-*T.gondii* ME49 antibodies (1 : 2,000) diluted with 1% bovine serum albumin (BSA) in tris-buffered saline with tween 20 (TBST) for one hour at room temperature. After a 30-minute incubation at room temperature, horseradish peroxidase (HRP)-conjugated goat anti-mouse immunoglobulin IgG produced an amplification signal. Visualization of this reaction was obtained by using 0.2% of 3,3-diaminobenzidine tetrahydrochloride (DAB). All DAB-treated tissue sections were stained with hematoxylin before being examined under a light microscope (Olympus, Hamburg, Germany) at 40, 100, and 400× magnification to stain the background.

### 2.8. Statistical Analysis

The means and standard deviations of quantitative data were calculated and compared using analysis of variance. The probability value of less than 0.05 was regarded as statistically significant. All calculations were performed using the Statistical Package for Social Science (SPSS), version 16. (Chicago, CA).

## 3. Results

### 3.1. Histopathological Study

#### 3.1.1. H&E-Stained Liver Smears

**Figure 1:**
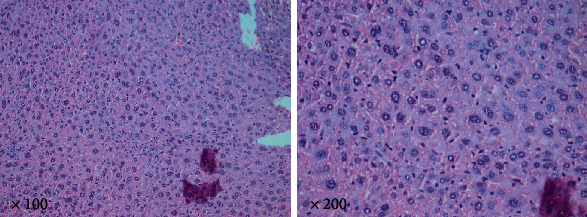
Control mice liver tissue showing normal hepatocytes (H&E. ×100 and ×200 magnifications).

The non-infected control group showed normal liver parenchyma with hepatocytes and portal tract in ill-defined typical hepatic lobules. Eosinophilic cytoplasm characterized the hepatocytes with prominent vesicular nucleoli **(**Figure 1**).**

#### 3.1.2. *T. gondii*–Infected Group

**Figure 2:**
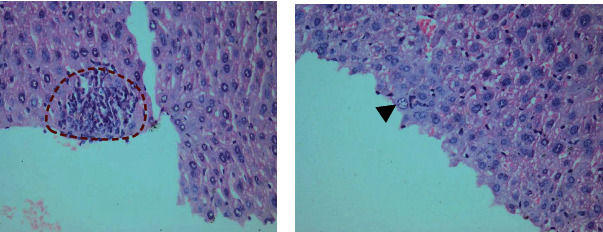
Infected Liver tissue showing (a) marked inflammatory cellular infiltrate (dashed line). (b) Liver ballooning (arrowhead) (H&E. ×400 magnification). Hepatocytes are showing vacuolated cytoplasm with vesicular nuclei.

**Figure 3:**
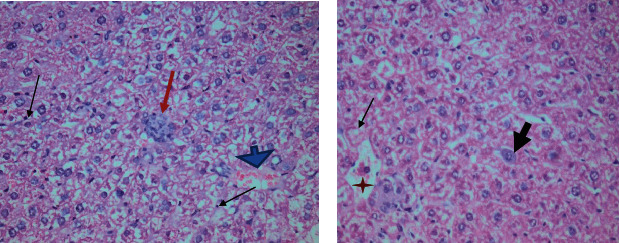
Liver tissue of *Toxoplasma gondii* chronically infected mice showing (a) intrahepatic image consistent with *Toxoplasma* cyst with bradyzoites (red arrow) with inflammatory cellular infiltrate (black arrows) and congested blood sinusoids (blue arrowhead). (b) Degenerated hepatic cells (big black arrow), some are devoid of nuclei (thin black arrow) and congested sinusoids (red star) (H&E. 400×).

**Figure 4:**
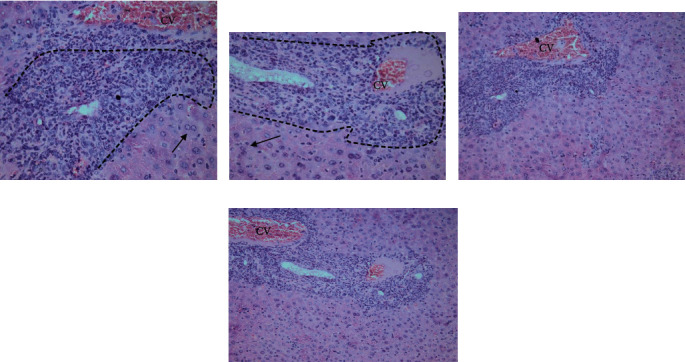
A micrograph of infected mouse liver showing marked inflammatory cellular infiltrate (dashed line) around the central vein (CV). Some bi-nucleated cells could be noted (black arrow) (H&E. Left (a, b): 400×, right (c, d): 200× original magnification).

Higher magnification of different zones of hepatic lobules showed marked vacuolated cytoplasm of swollen hepatocytes, disturbed sinusoidal architecture in between, and dilated central vein. Marked inflammatory infiltration around the portal tact was noted with sporadic small foci of inflammatory cells. Some blood sinusoids were dilated and showed significant congestion with interstitial hemorrhage. Some liver tissue sections showed bi-nucleated cells with few hepatocytes devoid of nuclei. Some liver sections also identified liver cell ballooning **(**Figures 2–4**).**

#### 3.1.3. H&E-Stained Spleen Sections

**Figure 5:**
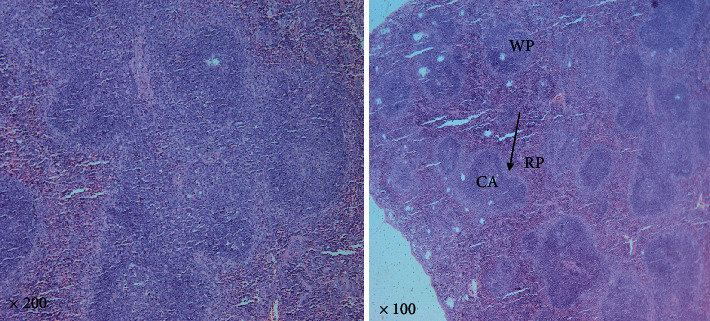
A photomicrograph of a control non-infected splenic section showing the normal architecture of the splenic pulps, which are the white (WP) and the red (RP) pulps. In the white pulp, the lymphatic follicles (arrows) and central arteriole (CA) are noticed. Red and white pulps were differentiated with a clear appearance of the trabeculae and marginal zone (H&E. 100× and 200× original magnification).

In the control non-infected group, spleen tissue sections from the control mice showed typical architecture of the parenchyma of the spleen (splenic pulp), which are the white and red pulps. The central arteriole occupies the white pulp. Lymphatic follicles containing lymphocytes were present. The lymphocytes were closely packed. The red pulp is demarcated from the white pulp by the marginal zone delimited from the lymphatic follicles by the marginal sinus. Cell cords networks called splenic cords constituted the red pulp and were separated by vascular sinuses with bulged nuclei (Figure 5).

#### 3.1.4. *T. gondii–*Infected Group

**Figure 6:**
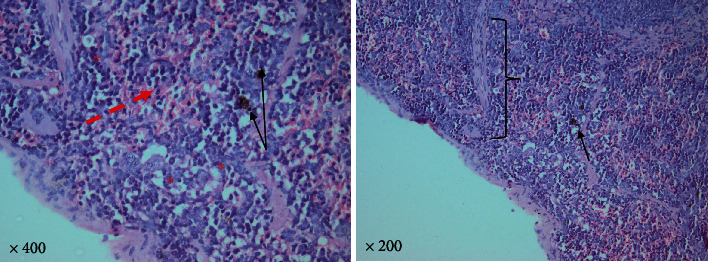
A photomicrograph of a mice splenic section (infected group) reveling loss of splenic architecture, markedly dilated blood sinusoids (dashed red arrow), and excess fibrinoid material in splenic trabeculae (brackets). Considerable hemosiderin pigments deposition was noticed (black arrow). Wide spaces (asterisks) and multiple cellular infiltrates are observed. Extravasation of blood is marked (dashed red arrow) (hematoxylin and eosin. 400× and 200× original magnification).

**Figure 7:**
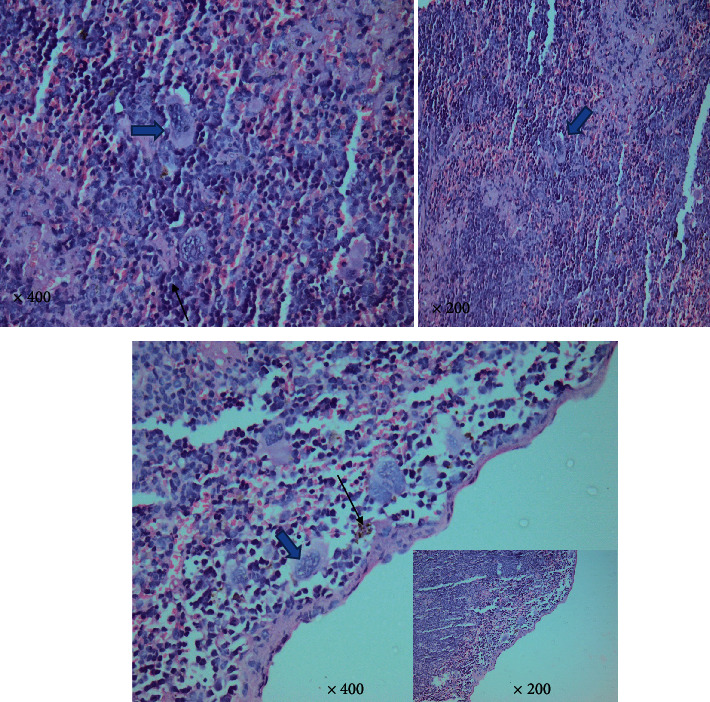
A photomicrograph of a mice splenic section (infected group) showing interrupted irregular splenic architecture with *T. gondii* cyst (big arrows). Hemosiderin pigments (thin black arrows). (H&E. 400× and 200× original magnification).

**Figure 8:**
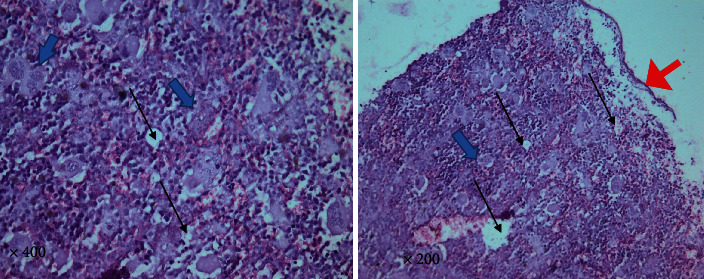
A photomicrograph of a mice splenic section (infected group) showing interrupted irregular splenic architecture with *T. gondii* cyst (big arrows) with marked cellular infiltrate. Significant extravasation of blood between multiple empty spaces is obvious (thin black arrows). Notice the thin shredded spleen capsule (red arrowhead) due to chronic *Toxoplasma* infection (H&E. Left: 400× and right: 200× original magnification).

Higher magnification of different spleen sections showed loss of splenic architecture, markedly dilated blood sinusoids, and fibrinoid material. The normal splenic arrangement was heavily interrupted with multiple *T. gondii* cysts surrounded with hallow. Hemosiderin pigments deposition was also noticed in some sections. Widening of blood sinusoids has been recorded with multiple cellular infiltrates in splenic tissue. Extravasation of blood was noticed (Figures 6–8).

#### 3.1.5. Immunohistochemical Results of Liver and Spleen Tissue

All tissues were reacted with antibodies against caspase-3, CD3, and CD138.

Immunohistochemistry (IHC) is a technique that uses an antigen-antibody response to visualize the extent and amount of a molecule's distribution in various tissues. It is carried out without destroying the histologic architecture of tissues, allowing the molecule's expression pattern to be seen in the context of the microenvironment. Caspase-3, CD3, and CD138 expression were found in varying intensities in the studied groups' liver and spleen tissues.

**Figure 9:**
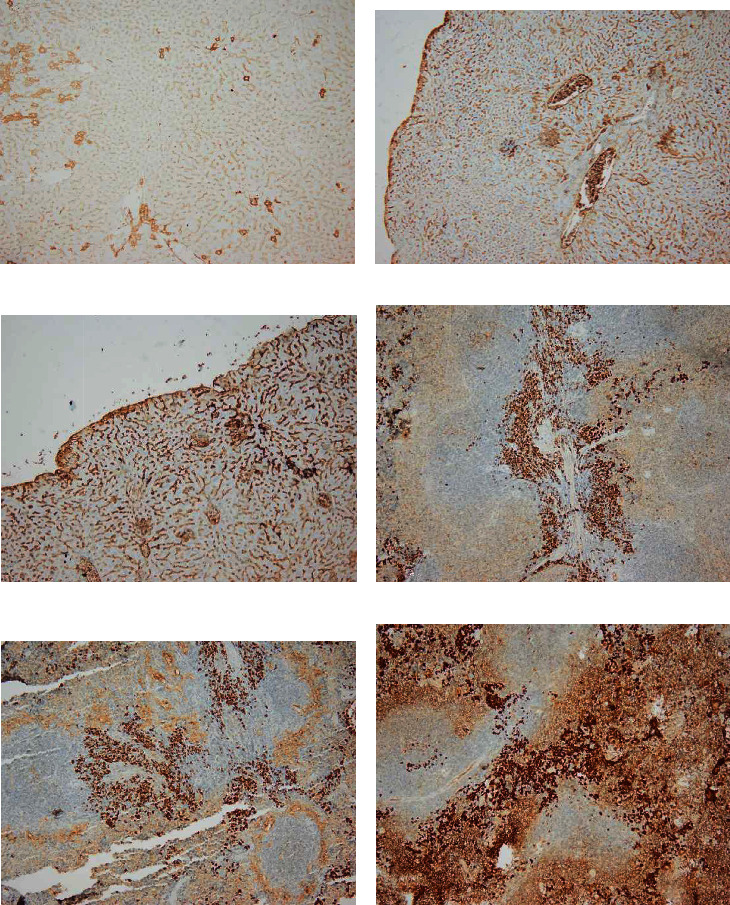
Photomicrographs of liver tissue (top row) and spleen (bottom row). The normal non-infected group showed (a) normal liver tissue (negative staining). The infected group showed (b) moderate inflammation and mild intensity, (c) mild inflammation and moderate positivity, (d) mild intensity and marked inflammation, (e) moderate positivity and marked inflammation, and (f) marked inflammation and strong positivity. Prominent cytoplasmic immune reactivity in the splenic cells of the white and red pulps is obvious. Because of cellular proliferation, the white pulp grew in size. The distinction between white and red pulp is significantly obscured. Dark pigmentation of most cells, while the spaces between sinusoids noticed markedly enlarged. The observed disarray may be caused by splenic lymphoid tissue hyperplasia. Inflammatory infiltration is evident in all sections of the infected group (caspase 3, ×100).

**Figure 10:**
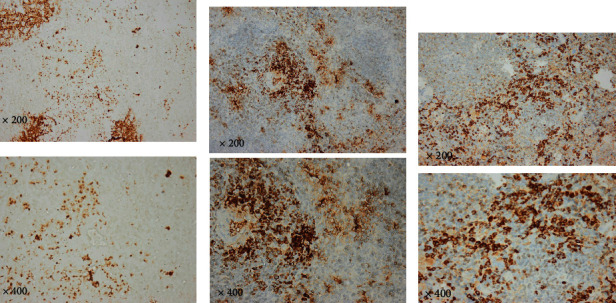
Photomicrographs of spleen tissue of infected group showing different inflammatory responses and different color intensities. (a) Moderate inflammation, mild intensity; (b) marked inflammation, moderate positivity; and (c) marked inflammation, strong positivity (CD3: spleen. Above row ×200 and down row ×400).

**Figure 11:**
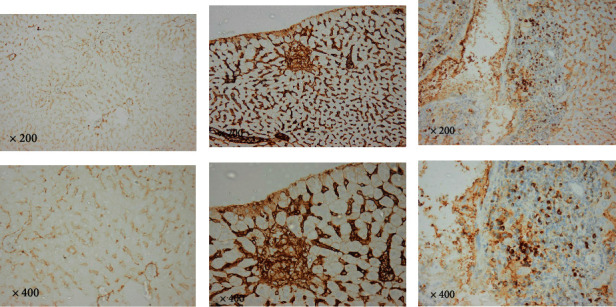
Photomicrographs of non-infected and infected liver tissue show different inflammatory responses and different color intensities. (a) Liver: no inflammation, no marker; (b) marked inflammation, strong positivity; and (c) marked inflammation, moderate positivity. (CD3: liver. Above row ×200 and down row ×400).

**Figure 12:**
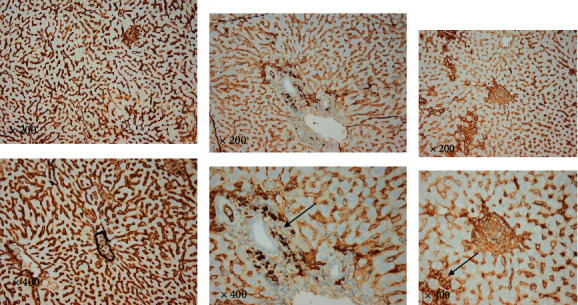
Photomicrographs of non-infected and infected liver tissue show different inflammatory responses and different color intensities. (a) Liver: no inflammation, no marker; (b) moderate plasma infiltration, strong positivity; and (c) moderate plasma infiltration, moderate intensity. (CD138: liver. Above row ×200 and down row ×400).

**Figure 13:**
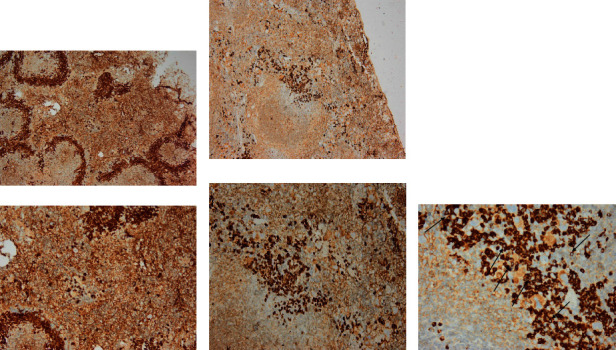
Photomicrographs of the non-infected and infected spleen tissue show different inflammatory responses and different color intensities. (a) Moderate plasma infiltration, strong positivity; (b) marked plasma infiltration, strong positivity; and (c) moderate plasma infiltration, strong positivity. Note cell membrane distribution of CD138 (arrows); **×**400. (CD138: spleen. Above row **×**200 and down row **×**400).

Caspase-3, CD3, and CD138 immunostaining reactions were negative in the liver and spleen tissues of the healthy control group (splenic data not published). The positive immunohistochemical reaction for all markers appeared as brown cytoplasmic staining. The liver and spleen tissue of the infected group showed a notable increase in caspase-3, CD3, and CD138 expression compared to the control non-infected group, indicating their upregulation during toxoplasmosis infection. Most of the sections showed different staining densities of the color of the markers (Figures 9–13).

### 3.2. Results of Serum Liver Biochemical Determinations and Nitric Oxide Level in Liver Homogenate

Table 1 displays the biochemical parameters of liver functions recorded in this study. *T. gondii* infection resulted in hepatotoxicity, as evidenced by elevated serum AST and ALT activity. A clear enzymatic levels difference in ALT, AST, TP, GGT, and AFP between infected mice and controls has been recorded.

The mean and standard deviation of nitric oxide (NO) levels in the liver tissue of mice infected with chronic toxoplasmosis and a non-infected control group are shown in Table 2. *T. gondii* infection increased nitric oxide (NO) levels in liver homogenates. There were significant changes in the level of nitric oxide (NO) content (*P* ≤ 0.05) in the liver homogenate of mice infected with chronic toxoplasmosis compared to the normal non-infected group.

## 4. Discussion

*T. gondii* is a protozoan parasite that invades mammalian cells by an actin-dependent mechanism leading to the formation of a parasitophorous vacuole (PV) which is primarily modified by the parasite. The parasite can affect any cell type of warm-blooded vertebrates [40]. *T. gondii* parasite has been isolated from many tissues other than the brain, including the liver, spleen, pancreas, bladder, lymph nodes, skin, and kidney [41]. Being an intracellular protozoon parasite, *T. gondii* secretes many virulence factors which act as master regulators of the host cell physiology to guarantee the intracellular survival of the parasite [42]. After the invasion, activation of an innate immune response is established in response to host cell invasion by the parasite. This reaction is followed by an adaptive immune response resulting in activation of the antigen-specific T and B cell types [43]. Unfortunately, this specific immune response is not aimed to get rid of the infection. Instead, it is directed toward the rapidly proliferating tachyzoites in the acute stage, evolving them to long-lived slow-growing bradyzoites in immune-evading cysts in the chronic stage. This reaction results in masking the disease; however, the infection remains dormant in various tissues, including the liver and spleen [44]. The major holes in our knowledge about *T. gondii* biology in terms of the mechanisms of cell-intrinsic immunity in different tissues have forced many researchers to investigate this mystery using rodent models, human cell experimental systems and make use of the available data from clinical cases [6]. In this respect, our study aims to draw attention to other organs affected by *T. gondii* parasites like the liver and spleen, which act as a potential source of biological markers to identify new diagnostic and therapeutic strategies against toxoplasmosis.

In this study, mice infected with the ME49 (cyst-forming type II) avirulent strain of *Toxoplasma* were allowed to survive through chronic infection and sacrificed 9-wpi. The pathological effect of toxoplasmosis on the liver and spleen during chronic illness was verified in immunohistochemical and histopathological sections.

The highly vascularized liver is a frequent target for foreign and self-antigens such as toxins, cell debris, various pathogens, and a permanent site of dysregulated inflammation [45]. This organ represents the second line of defense, which comes after the gut intestinal epithelial barrier, eliminating invading pathogens preventing their spread into the body [46]. The liver is well equipped with an army of well-primed transitional efficient cells and highly specialized, permanently resident, innate immune effectors in the form of Kupffer cells, hepatic stellate cells, and endothelial cells [45, 47]. This natural function can be interrupted by infections such as *T. gondii* that affect liver cells either independently or through the mechanism of upregulating pro-inflammatory cytokines, especially INF-*γ*, TNF-*α*, and IL-12 [48]. In *T. gondii* chronically infected mice, the reticuloendothelial cells' function in different tissues is also greatly altered by the infection [49] with subsequent induction of apoptosis following parasitic infiltration with tachyzoites of *T. gondii* inside the normal cells [50].

Histopathological findings in this study revealed inflammation in the liver of the toxoplasmosis-infected group compared to the control one that showed no histological alterations. Marked inflammatory infiltrations around the portal tract with many inflammatory foci were noticed. Some blood sinusoids were dilated and showed significant congestion with interstitial hemorrhage. Vascular congestion, necrosis, and cell lysis were also detected in some sections. Similar observations were recorded [51]. Hepatocytes tachyzoites were noticed with scattered hepatocellular necrotic foci and portal tract inflammation in 27^th^ day PI in mice liver. These histochemical changes become aggressive as the infection becomes more chronic. Hepatic ballooning degeneration had been reported in our work which was identified as markedly swollen hepatocytes. This change occurred in response to the massive influx of water and Na + because of ATP depletion and mitochondrial dysfunction that altered ion homeostasis and plasma membrane integrity [52]. In agreement with the present study, Fuentes-Castro et al. [53] reported changes in the hepatic structure with free tachyzoites in hepatic parenchyma (4 wpi) in mice infected with *T. gondii*. These changes can make headway to hepatomegaly, granuloma, hepatitis, and necrosis in infected hosts [54]. In another experimental study, both hepatocytes and stellate cells were found to be clustered with *Toxoplasma* parasite 6-day PI [55]. They linked the hepatic fibrosis observed in infected hosts to numerous activated hepatic stellate cells (HSCs), which were significantly higher in the infected toxoplasmosis group than in the healthy group. These cells are known to play an essential role in developing fibrosis and its progression to cirrhosis. Our histological findings are consistent with Sukthana *et al.* [56], who found tachyzoites and tissue cysts inside hepatocytes and sinusoidal liver capillaries.

Similarly, severe architectural disorder, inflammatory cell infiltration, notable fatty alteration, and fibrosis in *T. gondii*–infected liver sections were reported [57–59]. Notably, some researchers recorded negative results to *Toxoplasma* reproduction in hepatic cells [60]. After oral inoculation, *Toxoplasma* parasite either enters the mesenteric circulation then to the liver or travels through the lymphatic network [61]. When the parasite enters the liver, it proceeds progressively to the surface of sinusoidal epithelial cells, then to the Kupffer cells, and finally to the cytoplasm of hepatocytes [62]. This route may explain why the *Toxoplasma* parasite targets the liver during the early stages of infection [63] and affect other visceral organs like the spleen along the chronic course of the disease in the mammalian host [64]. As toxoplasmosis infection becomes more chronic, the intracellular tachyzoites increase rapidly over time and hepatocytes tissue cysts with those inhabiting the sinusoidal liver capillaries become abundant [51]. Melchor et al. [65] observed liver fibrosis in mice sacrificed 9 wpi. They attributed this result to the development of cachexia in their animal hosts in response to the inflammatory signals elevated in chronic *Toxoplasma* infection. This response is sufficient to induce cachexia over many weeks that lead to hepatic fibrosis in infected hosts.

The *T. gondii* parasite's ability to damage the liver may be owing to the parasite's mechanical action. Different research work [56, 66, 67] has hypothesized that parasite proliferation in the liver tissue may distort and disturb the metabolic activities of hepatic cells and injure their DNA, resulting in cell death. The parasite, on the other hand, could impair the liver indirectly through over-activation as well as increase levels of pro-inflammatory cytokines (INF-, TNF-, and IL-12) [54, 68].

The spleen serves as a second defense line against invading pathogens. Its unique morphological and functionally distinct parts (white and red pulp), aided by many specialized subsets of myeloid and dendritic cells, represent a dynamic cellular complexity targeting multiple infections [69]. The maintenance and organization of regular splenic compartments are controlled by complex immune responses disrupted by various parasitic diseases, including toxoplasmosis [70]. Mice infected with toxoplasmosis have reduced splenic CD8+ T and germinal center B cell responses because these immune cells are severely destroyed throughout the illness [71].

In the present work, H&E stained splenic cellular populations showed apparent damage and spatial alterations due to toxoplasmosis compared with standard control samples. White pulp typical structure has been disrupted, and the usual red pulp cellularity has been replaced with plasma cell infiltrate. We could detect *Toxoplasma* cyst in the splenic tissue interrupting its typical structure. This result was previously confirmed [72] by isolating tachyzoites of *T. gondii* from the spleen. In agreement with the present study, apparent alteration in spleen white pulp size and shape was described [73, 74]. Also, David et al. [49] confirmed that mice infected with chronic toxoplasmosis have sustained splenic and lymph node architecture changes. In line with these findings, it was stated that disruption of the white pulp structure of the spleen is a significant pathology of hemo-parasites such as *T. gondii* and is associated with splenomegaly, lymphoid tissue hyperplasia [75]. This infiltration was associated with the parasite dose; the higher the dose of infection, the higher the level of infiltration and apoptosis [50]. More histologic alterations like encroachment of red pulp compartment over white pulp area have been recorded [76]. On the contrary, Unno *et al.* [77] were unable to detect *T. gondii* parasite in the spleen, but the parasite can be found in other tissues such as the liver. One of the immunoregulatory mechanisms the *Toxoplasma* parasite uses to subvert host defenses and ensure intracellular dormancy in the chronic stage is change in mice splenic architecture associated with anti-parasitic humoral immune response dysregulated cytokines secretion [74]. These structural abnormalities may significantly impact the spleen tissue's and other tissues' metabolic microenvironment [78]. As a result, we could hypothesize that understanding this interactive relationship is vital for developing medications that target parasite survival pathways during their latent stages [76].

The immunohistochemistry (IHC) technique is known for its accuracy in labeling and locating the distribution and amount of a specific molecule in cells and tissues using specific antigen-antibody reactions [79]. It is carried out without destroying histologic architecture, so assessing an expression pattern of any molecule is possible in respect of the microenvironment [80]. In the present study, we analyzed spleen and liver tissues of both working groups using specific surface markers, anti-caspase 3, anti-CD3, and anti-CD138, by immunohistochemistry.

Caspase-3 is an important molecule involved in programmed cell death (apoptosis) through induction, transduction, and amplification of intracellular apoptotic signals. It is present in an inactive form inside the cell (procaspase phase) until activated by a series of biochemical changes [81]. Apoptosis is one of the host defense mechanisms used to get rid of intracellular infections [82, 83]. However, during *Toxoplasma* infection, the parasite is resistant to multiple apoptosis inducers. So, it cannot be easily eliminated by the natural death of the infected host cell (apoptosis) or by T cell-mediated cytotoxicity [84, 85]. Indeed, *T. gondii* can inhibit caspase activation with the subsequent abortion of apoptosis in the murine host [86] and different human cell lines [31, 87] from preserving its intracellular niche for sustainable, productive infection.

In contrast to the previous findings, the immunohistochemical examination of liver and spleen sections in the present work revealed positive caspase-3 immunoreactivity in the cytoplasm of the hepatocytes and splenic cells. In line with the present finding, some investigators reported apoptotic response in murine splenic cells following *T. gondii* tachyzoite infection [50]. Caspase-3 was significantly upregulated in the brain of mice after *T. gondii* ME49 contributing to neurological pathology [88]. In a concentration-dependent manner, *T. gondii* tachyzoite inhibited the proliferation of hepatic carcinoma cell through upregulation of caspase-3 promoting its apoptosis [89]. Amazingly, caspase's activation results in the cleavage of many proteins related to the cell's structural integrity, resulting in the cell's destruction [90]. Apoptosis in the spleen causes immune system disturbances [91]. In the present work, we could attribute failed cell death in the presence of caspase-3 positive immunoreactivity to the non-apoptotic actions of caspase-3. These actions are obvious in the compensatory proliferation repair phase (a phase evident in our work by binucleated cells which considered as a sign of regeneration), during which caspases play a functional role in cell growth control and provide the ultimate shape and size to tissues, allowing them to regain their previous cell numbers [92].

CD3 (cluster of differentiation 3) protein complex is involved in the activation of both cytotoxic T cells (CD8+ naive T cells) and T helper cells (CD4+ naive T cells). This antigen was identified as uniquely attached to the membranes of all mature T-cells and present at all phases of T-cell maturation, making it an ideal T-cell marker [25]. In the current work, immunoreactivity to this molecule was evident in the infected liver and spleen murine tissues compared to the healthy non-infected control. This indicates a strong T cell response to the parasite during dormancy. In comparison to our findings, cytotoxic CD3 + CD8+ T cells were found in more significant numbers in murine splenic tissue and peripheral blood of chronically infected mice [32]. Parasites are thought to continuously be discharged from cysts in chronically infected hosts, resulting in a permanent boosting of the immune system. T cell populations are critical for the long-term protection and to keep the infection in a chronic state protecting the host from tachyzoite development and subsequent disease [93]. T-lymphocytes, specifically CD4+ (helper) T-cells and CD8+ (cytotoxic) T-cells, are vital participants in established resistance. CD4 T cells function as support cells for infection-fighting CD8 T cells [94]. This role was proved by the results of T-cell depletion in different hosts that caused the infection to reactivate quickly with rapid tachyzoite growth, leading to fulminant pathology and mortality [95–97]. Surprisingly, the functionality of the T cell population is found to be an essential determinant in keeping the infection in the chronic phase than the number. During the later stages of chronic toxoplasmosis, CD8 T lymphocytes gradually express inhibitory receptor PD-1, leading to fatigue and dysfunctionality [97]. As a result, maintaining a sufficient level of T cell activity may be an important therapeutic strategy for treating chronic toxoplasmosis and preventing the reactivation of latent infections.

One of the most abundant proteins on the surface of plasma cells is syndecan-1, or CD138. CD138 stains only plasma cells and lacks its expression in most inflammatory cells. An increased population of plasma cells, evident by positive immunoreactivity to (CD138+) in the spleens and livers of chronically infected mice, has been recorded in our study compared to the control non-infected group. These findings imply that *T. gondii* tachyzoites cause an immunological response by activating plasma cells. A considerable increase in the expression of CD138+ plasma cells in the spleens of chronically infected mice was found [32], which is consistent with our findings. These plasma cells were in charge of maintaining long-term particular antibody levels in the blood.

On the other hand, the level of expression of this molecule was increasingly reduced in the chronic phase of infection from the 7th to 12th weeks in brain tissue of mice infected with toxoplasmosis compared to non-infected control [98]. CD138 (syndecan-1), which is increased during antibody-secreting cells (ASCs) maturation, is needed for mounting an efficient long-term humoral immune response by promoting survival of ASCs in a cell-intrinsic manner [99]. However, the precise role of CD138+ in toxoplasmosis has yet to be determined. According to our finding, the notable rise in antibody-secreting CD138 cells (plasma cells) in conjunction with activation of cellular immunity represents a collaboration of diverse immunological pathways for toxoplasmosis immune defense.

When pathological processes are induced in experimental animals, laboratory tests become an excellent tool to evaluate the physiological and functional changes in the affected organ. Thus, the biochemical profile in terms of liver enzymes was analyzed to see whether these histological changes altered liver function or not. Because ALT is primarily present in hepatocyte mitochondria and has a longer half-life, its concentration is preferable in measuring liver cell integrity versus other enzymes [100]. However, the concomitant increase in their serum values is still an excellent marker of hepatocellular injury. The liver is the primary nutrient absorption, metabolism, and storage regulator. So, any cellular damage and dysfunction of this organ will leak biochemical substances such as liver enzymes into the blood circulatory system [101]. Therefore, a high level of biochemical values in blood indicates abnormal liver function. Compared to the control group, our study found that toxoplasmosis had a substantial effect on liver function test enzymes in infected subjects. Similarly, a remarkable increase in reading values of liver enzymes in sera of studied mice after infection was reported [102]^,^ which reversed to normal after treatment. El-Sayad et al. [51] affirmed that as toxoplasmosis infection duration increased, so did the reduction of enzyme function. This finding coincided with discovering many parasites and necrosis within liver tissue. *T. gondii* enters a dormant condition during chronic infection; yet, even the dormant parasite stage may significantly impact the host metabolic profile [76]. This metabolic function dramatically affects the activity of the immune system [103]. Al-Kuraishy *et al.* [104] proposed that elevated ALT and AST values could be the earliest sign of hepatic injury caused by toxoplasmosis, as seen in cytotoxic and cholestatic hepatic injuries.

Being an intracellular pathogen, *Toxoplasma* stimulates the increased production of pro-inflammatory mediators such as IL-12, IFN-*γ*, and nitric oxide, which characterize Th1 cytokine profiles [16]. The enzyme inducible nitric oxide synthase (iNOS) is responsible for the L-Arginine mediated NO production. Different cells upregulate the synthesis of this enzyme, resulting in the production of Nitric oxide (NO) in response to cytokine stimulation, limiting the replication of *T. gondii* inside the infected cell [105]. IFN-*γ* activated murine macrophages [106] and inflammatory monocytes [43, 107] are among the cells implicated in NO synthesis that exhibit nitration, oxidation, and chlorination reactions against invading microorganisms, including *Toxoplasma* parasite. The role of this enzyme in toxoplasmosis was evident by increased parasite burdens in infected mice administered iNOS inhibitor aminoguanidine and increased susceptibility to toxoplasmosis in iNOS-deficient mice during the late phase of infection [16]. Increased NO level during toxoplasmosis is pathognomonic, which is generated to control the disease [108]. However, all strains of toxoplasmosis adopted an evading strategy to escape NO harmful effect. They inhibit NO production in activated macrophages by degrading iNOS expression by activating potent antioxidant enzymes [106, 109]. Most of the literature's research has focused on describing oxidative stress during the acute phase of toxoplasmosis. However, many works investigating the oxidant-antioxidant balance in the later stages of *T. gondii* infection are still defective. In the present work, a significant rise of nitric oxide level has been recorded in liver homogenate of a chronically infected group of mice sacrificed 9 wpi compared with the non-infected control subjects. This result is not surprising since *Toxoplasma* infection is known to induce oxidative stress in the liver of infected hosts [108]. This stressful condition is a state of imbalance between the production of reactive oxygen species (ROS) by the infected host and the antioxidant system of the invading organism [110]. In an experiment where mice sacrificed earlier than our time (10- and 30-days PI), high levels of nitric oxide were remarkably recorded in the brain of *T. gondii*–infected mice than in the uninfected control group [111]. In agreement with our observation, liver tissues from the infected untreated group sacrificed eight weeks PI revealed strong iNOS expression, indicating increased NO levels in this tissue. This finding was associated with significant histopathological insult in liver parenchyma [59]. It has been reported that the pathophysiology of toxoplasmosis is related to the increased risk of oxidative stress in host cells due to the activation of inflammatory response against the parasite [104]. Alajmi et al. [58] recorded higher amounts of NO in toxoplasmosis-inoculated mice's liver homogenate, as well as a significant decrease in the activity of toxoplasmosis antioxidant enzymes. This elevation was significantly reduced following successful treatment.

NO generation at high levels during the early stages of acute infection can kill intracellular parasites and prevent *T. gondii* reproduction in macrophages and other cell types [112, 113]. Furthermore, in the late acute stage, a critical signaling molecule initiates the conversion of tachyzoite to bradyzoite forms [114, 115]. Its role in the late stages of infection is still being researched. However, the static effect of NO on *T. gondii* replication could be explained according to studies that used intracellular bacterial pathogens and have stated that NO can impede bacterial enzymatic activity and directly damage its DNA [116]. On the other hand, excess amounts of NO contribute to oxidative injury, leading to injury to host cells and subsequent disease pathology development [58, 117]. Chronically *T. gondii*–infected mice continuously show an elevated level of NO production the increased expression of IFN-*γ* because of the ongoing chronic infection releasing *T. gondii* antigens from semi-dormant tissue cysts that persist in different tissues [118]. This generated oxidative stress leads to intracellular lysosomal membrane damage, which is followed by apoptosis or necrosis [119]. So, it is hypothesized that any chemical compound that affects the oxidant-antioxidant balance will be a potent and novel anti-*Toxoplasma* therapeutic agent.

## 5. Conclusions

The liver and spleen are among the primary targets for toxoplasmosis in the late stages. The parasite exhibited an unfavorable inflammatory effect on chronically infected mice's liver and spleen tissues, characterized by altered histological features accompanied by an inflammatory reaction. The hepatic biochemical profile showed increased liver enzymes, and induced oxidative stress, evident by elevated NO concentration in liver homogenate. Levels of caspase3, CD3, and CD138 are markedly expressed by liver and spleen tissues compared to normal. These findings indicate that latent *T. gondii* stages can affect the host's metabolic and immunological profile creating a persistent challenging state between the parasite and the host. So, a better understanding of the expression of different molecules during toxoplasmosis, particularly during the chronic stage, is critical to identify potential therapeutic approaches against this infection.

## Figures and Tables

**Table 1:** Mean and standard deviation of serum level of liver biochemical activities in infected and control studied groups.

Group		ALT	AST	TP	GGT	AFP
1	Control group	49.1 ± 3.03	41.7 ± 1.76	6.91 ± 0.56	3.7 ± 0.22	6.2 ± 0.04
2	Infected group	73.9 ± 5.25	68 ± 2.66	8.07 ± 0.49	5.4 ± 0.11	7.6 ± 0.07

Alanine aminotransferase (ALT), aspartate aminotransferase (AST), gamma-glutamyl transferase (GGT), thymidine phosphorylase (TP), and alpha fetoprotein (AFP).

**Table 2:** Changes in the liver tissue level of NO content of mice infected with chronic toxoplasmosis and non-infected groups.

Group		*N*	Mean	SD	*t*-test	*P* value
1	Control group	10	25.3	2.05	23.17	≤0.05∗
2	Infected group	10	74.7	6.54		

∗Significant: *P* ≤ 0.05.

## Data Availability

Data available on request through contacting the corresponding author Dr. Mohamed Sarhan (email: drsarhan@zu.edu.eg; drsarhan@gmail.com).
